# National centralization of Hirschsprung’s disease in Sweden: a comparison of postoperative outcome

**DOI:** 10.1007/s00383-024-05842-6

**Published:** 2024-10-05

**Authors:** Linnea Söderström, Christina Graneli, Daniel Rossi, Kristine Hagelsteen, Anna Gunnarsdottir, Jenny Oddsberg, Pär-Johan Svensson, Helena Borg, Matilda Bräutigam, Elisabet Gustafson, Anna Löf Granström, Pernilla Stenström, Tomas Wester

**Affiliations:** 1 Department of Women’s and Children’s Health, Karolinska Institutet, Stockholm, Sweden; 2 Department of Pediatric Surgery, Skåne University Hospital Lund, Lund, Sweden; 3 Department of Pediatrics, Clinical Sciences, Lund University, Lund, Sweden; 4 Unit of Pediatric Surgery, Karolinska University Hospital, S3:02, 171 76 Stockholm, Sweden; 5 Department of Pediatric Surgery, Queen Silvia’s Children’s Hospital, Gothenburg, Sweden; 6 Department of Pediatric Surgery, University Children’s Hospital, Uppsala, Sweden

**Keywords:** Centralization, Postoperative, Surgical outcome, Hirschsprung’s disease, National specialized medical care

## Abstract

**Background:**

In Sweden, surgical treatment of Hirschsprung’s disease (HSCR) was centralized from four to two pediatric surgery centers 1st of July 2018. In adults, centralization of surgical care for complex or rare diseases seems to improve quality of care. There is little evidence supporting centralization of pediatric surgical care. The aim of this study was to assess surgical management and postoperative outcome in HSCR patients following centralization of care.

**Methods:**

This study retrospectively analyzed data of patients with HSCR that had undergone pull-through at a pediatric surgery center in Sweden from 1st of July 2013 to 30th of June 2023. Patients managed from 1st of July 2013 to 30th of June 2018 (before centralization) were compared with patients managed from 1st of July 2018 to 30th of June 2023 (after centralization) regarding surgical treatment, unplanned procedures under general anesthesia or readmissions up to 90 days after pull-through as well as complications classified according to Clavien–Madadi up to 30 days after pull-through.

**Results:**

In the 5-year period prior to centralization, 114 individuals from 4 treating centers were included and compared to 83 patients from 2 treating centers in the second period. There was no difference regarding age at pull-through or proportion of patients with a stoma prior to pull-through. An increase of laparoscopically assisted endorectal pull-through (8.8% to 39.8%) was observed (*p* < 0.001). No significant differences were seen in postoperative hospital stay, unplanned procedures under general anesthesia, or readmissions up to 90 days after pull-through. There was no difference in severe complications (Clavien–Madadi ≥ 3); however, HAEC treated with antibiotics increased following centralization (10.5–24.1%; *p* = 0.018).

**Conclusion:**

Centralization of care for HSCR does not seem to delay time to pull-through nor reduce severe complications, unplanned procedures under general anesthesia or readmissions up to 90 days after pull-through. The increased HAEC rate may be due to increased awareness of mild HAEC.

Level of evidence: Level III.

## Introduction

Hirschsprung’s disease (HSCR) is a congenital anomaly of the distal hindgut, affecting the enteric nervous system and presenting with an aganglionic segment in the distal colon with various extent to proximal bowel [1]. HSCR is a rare disease, affecting 1:5000 live births with a 4:1 male to female ratio [1]. The condition requires surgical intervention, so called pull-through, usually within the first year of life. The goal of surgery is to resect the aganglionic segment and form an anastomosis between healthy ganglionic bowel and the anus, preserving the external anal sphincter [1, 2].

In a few European countries, highly specialized pediatric surgical care is centralized to only some selected centers [3, 4]. In Sweden, the National Board of Health and welfare decided to centralize the care of multiple pediatric surgical diagnoses from July 2018, according to the process of process of Specialized Medical Care. This included care of patients with esophageal atresia, congenital diaphragmatic hernia, anorectal malformations, bladder exstrophy, and HSCR. Currently, two treating centers surgically manage HSCR patients, Karolinska University Hospital, Stockholm and Skåne University Hospital, Lund, as compared to four active centers prior to centralization.

Evidence suggests that centralization of surgical care for rare or complex diseases benefits adult patients, reducing both mortality and morbidity. Hospital volume, surgeon volume, and “effects of specialization” with the ability to provide dedicated training at expert centers have been proposed as the three predominantly influencing elements of the positive outcome. Furthermore, “failure-to-rescue” patients with postoperative complications at non-index hospitals may contribute [5]. Pediatric surgery is, by nature, a specialty of rare cases and low numbers seemingly favoring the idea of centralized care. However, studies on centralization of pediatric surgical care are scarce. A few studies have shown positive effects following centralization of portoenterostomy in biliary atresia [6–8]. In addition, centralization of surgical care for neuroblastoma and liver tumors seems to result in improved outcome [9, 10].

Recent data comparing the preoperative course of HSCR patients at the Karolinska University Hospital following centralization suggest that despite increasing patient volumes and longer time from diagnosis to pull through, centralization of care for HSCR does not seem to change the preoperative management and risk of complications [11]. There are no studies evaluating the surgical management and postoperative outcomes following centralization. Thus, the aim of this study was to assess surgical management and postoperative outcome in HSCR patients following centralization of care in Sweden.

## Methods

### Study design

This was a retrospective observational study.

### Study population

Inclusion criteria targeted all patients that had undergone pull-through surgery at one of the pediatric surgery center in Sweden from 1st of July 2013 to 30th of June 2023. Patients were identified from the hospitals’ digital case record system. Participants were divided into two groups based on time for surgery with a 5-year time period before and after centralization 1st of July 2018. Re-do pull-throughs during the time period were not considered.

### Participating centers

The participating centers were: Karolinska University Hospital, Stockholm; Skåne University Hospital, Lund; University Children’s Hospital, Uppsala; and Queen Silvia’s Children’s Hospital, Gothenburg. All statistical analyses were performed at the coordinating site at Karolinska University Hospital.

### Data collection and measurements

Data were collected retrospectively through review of digital medical records. Demographic variables included sex, birth weight, gestational age, familial disease, presence of other congenital malformations or syndromes, genetic abnormalities and place of birth. For gestational age, full-term was defined as the period from 37 to 41 weeks (preterm < 37 weeks; post term ≥ 42 weeks). Registered preoperative and surgical data included but were not limited to: age at first rectal biopsy, re-biopsy, age at definitive histopathology report, need for stoma, length of aganglionic segment, surgical technique, length of hospital stay. Unplanned readmissions and unplanned surgical procedures under general anesthesia up to 90 days following pull-through were considered. Postoperative complications within 30 days were graded according to the Clavien–Madadi classification [12]. Only complications grade ≥ 3 were considered. The incidence of Hirschsprung’s associated enterocolitis (HAEC) was based on information extracted from the case notes. A HAEC episode was defined as high-index suspicion by treating physician resulting in treatment with antibiotics.

### Statistical analysis

Statistical analysis was performed using IBM SPSS (version 29.0.1.0). Data are presented as percentages for categorical variables as well as median and range for numerical variables. Chi-square was used for comparative statistics of categorical data when applicable and Fishers exact test when assumptions of Chi-square were violated. For numerical data, Mann–Whitney *U* test was used when comparing two independent groups. Statistical significance was set at *p* ≤ 0.05.

### Ethics

This study was approved by the Swedish Ethical Review Authorities, registration number 2023-02650. All procedures were in accordance with the Helsinki declaration and its later amendments.

## Results

### Study population

**Fig. 1:**
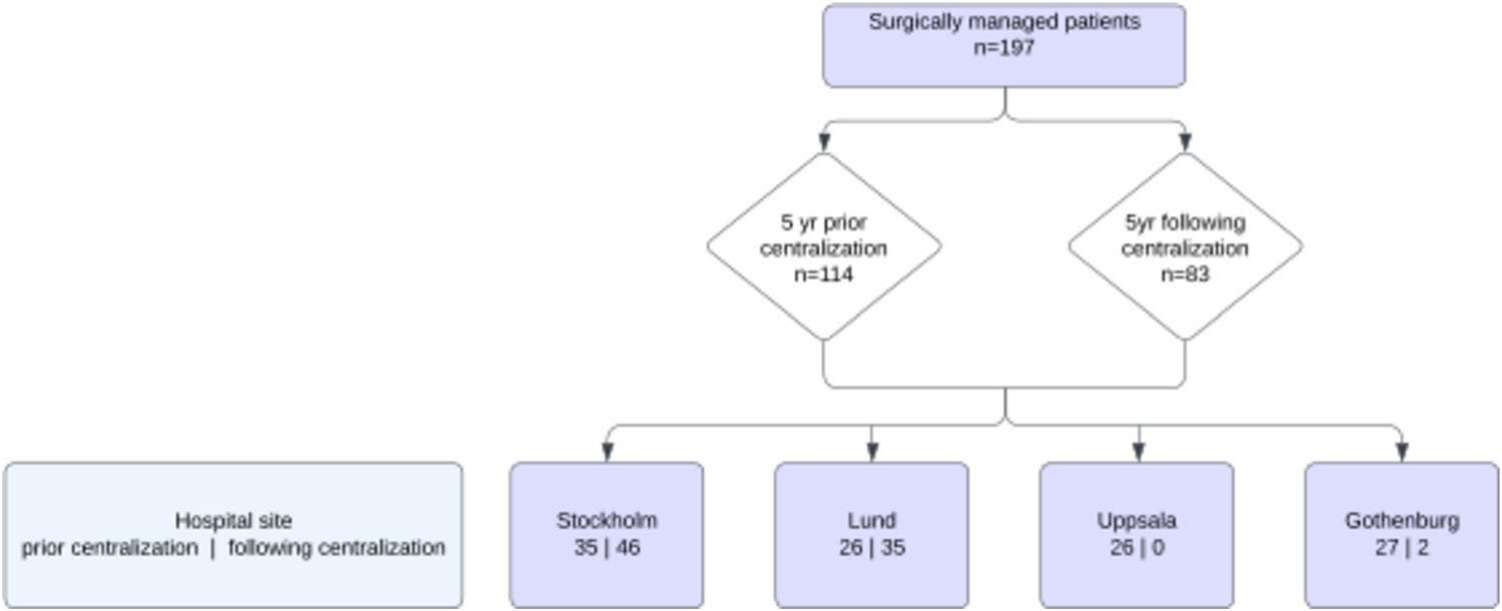
Site of surgical treatment prior to and following centralization

A total of 197 individuals met the inclusion criteria of being surgically managed at one of the participating centers between 1st of July 2013 to 30th of June 2023 and were included in the study. During the 5 years prior to centralization, 114 individuals had undergone pull-though compared to 83 during the 5 years following centralization. All but two patients were managed at either Karolinska University Hospitals or Skåne University hospital following centralization (Fig. 1).

#### Patient characteristics

Demographic features of HCSR patients prior to and following centralization are depicted in Table 1. Comparing the two time periods, no significant differences were found between the groups concerning sex, gestational age, birth weight, familial disease, associated malformations and/or syndromes, and type of HSCR (rectosigmoid, long segment, or total colonic aganglionosis).

**Table 1:** Demographic features

Patient characteristics	Prior to centralization *n* = 114	Following centralization *n* = 83	p value
Sex, *n* (%)			0.858
Male	92 (80.7)	66 (79.5)	
Female	22 (19.3)	17 (20.5)	
Gestational age, *n* (%)			0.270
Pre-term	11 (9.6)	5 (6.0)	
Term	87 (76.3)	69 (83.1)	
Post-term	9 (7.9)	2 (2.4)	
Unknown	7 (6.1)	7 (8.4)	
Birth weight, g, median (min–max)	3550 (1854–4712)	3632.5 (1887–4750)	0.445
Familial disease, *n* (%)	14 (12.3)	10 (12)	1.00
Associated malformations and/or syndromes, *n* (%)	23 (20.2)	11 (13.3)	0.253
Trisomy 21	10 (8.8)	6 (7.2)	
Other syndrome	5 (4.4)	3 (3.6)	
Type of HSCR, *n* (%)			0.223
Rectosigmoid	98 (86.0)	66 (79.5)	
Long segment	9 (7.9)	13 (15.7)	
Total colonic aganglionosis	6 (5.3)	3 (3.6)	
Unknown	1 (0.9)	1 (1.2)	

### Outcomes

There were no significant differences regarding the diagnostic procedures for HSCR patients prior to and following centralization (Table 2). The prevalence of patients that needed a stoma prior to pull through did not change. The most common type of surgical treatment during the time period was total transanal endorectal pull through, however, following centralization the proportion of laparoscopically assisted endorectal pull-through increased. Six patients with total colonic aganglionosis underwent endorectal pull-through and three open Duhamel. Two patients prior to centralization that initially underwent total transanal pull-through were misdiagnosed as having a short segment and both underwent revisions later on.

**Table 2:** Diagnostics and surgical treatment

	Prior to centralization *n* = 114	Following centralization *n* = 83	*p* value
Age at biopsy, days, median (min–max)	6.5 (1–2000)	8 (1–1623)	0.640
Re-biopsy, *n* (%)	25 (21.9)	18 (21.7)	0.600
Age at diagnosis, days, median (min–max)	32 (2–2039)	24 (4–1636)	0.109
Preoperative stoma, *n* (%)	19 (16.7)	22 (26.5)	0.111
Time from diagnosis to surgery, days, median (min–max)	33 (0–1021)	31.5 (0–960)	0.569
Age at pull through, days, median (min–max)	76 (8–2231)	66 (10–1964)	0.149
Surgical treatment *n* (%)			** < 0.001**
Rectosigmoid and long segment disease			
Total transanal endorectal pull through, with or without subumbilical incision for biopsies	71 (62.3)	36 (43.4)	
Endorectal pull through, with laparotomy	23 (20.2)	10 (12)	
Endorectal pull through, laparoscopy assisted	10 (8.8)	33 (39.8)	
Endorectal pull through, robot-assisted	1 (0.9)	0	
Duhamel (open)	0	1 (1.2)	
Unknown	3 (2.6)	0	
Total colonic aganglionosis			
Duhamel (open)	1 (0.9)	2 (2.4)	
Endorectal pull through*	4 (4.4)	1 (1.2)	

*Including two patients initially misdiagnosed as rectosigmoid disease that later underwent revision

Statistically significant values are in bold (*p* < 0.05)

Following pull-through, the median time to discharge was 5 days during both time periods (Table 3). No differences were observed in unplanned readmissions or unplanned surgical procedures under general anesthesia in the first 90 days after pull-through. Prevalence of complications (Clavien–Madadi ≥ 3) in the first 30 days following pull-through did not differ between the time periods (*p* = 0.177) nor severity of complications (*p* = 0.353). There were no grade IV or V complications registered. Only one HAEC case, prior to centralization, was graded as Clavien–Madadi 3b and included in the analysis. Postoperative HAEC requiring treatment with antibiotics in the first 90 days following pull-though increased from 10.5% to 24.1% following centralization (*p* = 0.018). One death occurred during the time period. The child was found deceased at home and no conclusive cause of death was found.

**Table 3:** Postoperative outcome

	Prior to centralization *n* = 114	Following centralization *n* = 83	*p* value
Postop hospital stay, days, median (min–max)	5 (2–42)	5 (2–34)	0.224
Complications Clavien–Madadi (≥ 3)			0.177
(30d), n (%)	6 (5.3)	9 (10.8)	
IIIa	1 (0.9)	5 (6)	
IIIb	5 (4.4)	4 (4.8)	
HAEC (90d) n (%)	12 (10.5)	20 (24.1)	**0.018**
Unplanned readmission (90d) n (%)	18 (15.8)	17 (20.5)	0.354
Unplanned surgical procedure (90d) n (%)	14 (12.3)	14 (16.9)	0.449
Death n (%)	1 (0.9)	0	1.00

Comparing patients at the centers providing the national specialized medical care (i.e., Stockholm and Lund) prior to and following centralization showed no significant differences in any of the variables in Table 3.

## Discussion

### Key findings

In 2018, surgical management of HSCR was centralized from four to two centers in Sweden. This retrospective, multi-center study aimed to assess the early postoperative outcomes following centralization of care for HSCR. Surgical management shifted, with an increase of laparoscopically assisted endorectal pull-through (8.8% to 39.8%). No significant differences were seen in postoperative hospital stay, unplanned procedures under general anesthesia or readmissions up to 90 days after pull-through. Severe complications (Clavien–Madadi ≥ 3) up to 30 days after pull-through did not differ between the time periods. HAEC treated with antibiotics increased following centralization (10.5% to 24.1%).

### Interpretation

From initial diagnostics to early postoperative outcome, few changes were observed following centralization. There was a 27% reduction in patients from 114 in the 5 years prior to centralization to 83 in the 5 years following centralization. This might partially be accounted for by declining birth rates and a higher proportion of children being born in the spring and summer months. From 2013 to 2018, a mean of 115 341 births per year was recorded compared to 110 413 births per year 2018 to 2023. Birth rates in 2023 were the lowest recorded during the time period, only 100 051 [13]. Furthermore, some patients born in 2023 with late diagnosis or postponed surgical treatment may have been excluded, as only patients who had undergone definitive repair the 30th of June were included.

There were no differences in age at biopsy, need of re-biopsy, age at diagnosis or time from diagnosis to pull-through between the time periods. With fewer centers, the travel distance to treating hospital increased for many patients following centralization. There are studies indicating a higher risk of delay in diagnosis and misdiagnosis in patients residing at longer distance to the tertiary referral hospital. This concern may be even higher in patients with rare diseases [14, 15]. Therefore, it is crucial that our results do not indicate such disparities, ensuring accurate and timely diagnoses despite patients living more distant to the referral hospital.

Surgical technique differed prior to and following centralization. The most noteworthy being an increase in laparoscopically assisted pull-through from 8.8% to 39.8%. Trananal endorectal pull-through remained the most commonly performed procedure, 65.8 and 43.4%, prior to and following centralization. Although these findings are interesting and probably reflects a change in the general trend of HSCR treatment in Sweden, it seems unlikely to have been an effect of centralization nor impact the results as all procedures are considered feasible and safe without evidence supporting superiority in outcome for either [16].

Although volume-outcome benefit for complex surgery is widely recognized [17–19], results of our study did not show a reduction of postoperative complications, unplanned procedures or unplanned readmissions following centralization of care. Many studies favoring centralization consider high-risk procedures with considerable short-term morbidity as well as complex procedures for conditions with high mortality. In high-income countries, HSCR mortality rates are low, while long-term morbidity remains a pressing issue [20–23]. This may reflect why our results, considering only short-term outcome, does not show any significant improvement in outcome. The number of patients managed in each center is low also after centralization, which is important to take into account when the results are interpreted. It could be argued that the potential advantages of centralization require larger volumes.

HAEC requiring treatment with antibiotics increased from 10.5 to 24.1%. Although noteworthy, this rise might be attributed to increased awareness of mild HAEC cases managed at home with oral antibiotics and transanal irrigations. There does not seem to be differences with respect to incidence rate of residual aganglionosis or anastomotic strictures, that could potentially explain the increased HAEC incidence. We have previously shown at our center that at-home transanal irrigations, although utilized during the preoperative course, remains a safe and adequate mode of decompression despite living far from the index hospital [11]. In addition, the threshold for initiating treatment might be lower in patients who do not have easy access to the index hospital, as a form of safety precaution. Unfortunately, we cannot determine the true cause of the reported increase in HAEC, as no grading of severity or symptoms was conducted.

### Limitations

Results of this study should be considered with regards to several limitation. The retrospective design of the study may impact the accuracy of the collected variables. Since multiple individuals collected the data, there is a risk of inconsistent interpretation of reviewed variables. In addition, the limited size of the HSCR cohort affects the study’s power and increases the risk of type II errors. Comparing patients from two different time periods and different hospitals may also introduce confounding factors, such as changes in senior staff, new procedures or protocols, and evolving scientific evidence influencing treatment practices.

### Generalizability and clinical implications

This is the first study of its kind evaluating the surgical and postoperative course of patients with HSCR following centralization of care. It provides a national oversight of patient outcome following this change of procedure in Sweden and is one of few existing studies evaluating centralization of pediatric surgery.

While our study provides valuable information on the topic of centralization, results should be interpreted cautiously when applied elsewhere. Diagnostic methods and treatment protocols may vary significantly from one country to another. The unique demographic and organizational aspects of Swedish healthcare may also limit the generalizability of the results to other settings. In addition, the implementation of centralization can differ across countries, influencing caseloads, travel distances, and outpatient management. In countries where the number of pediatric surgery centers is high relative to the population, with some centers potentially handling a very limited number of cases per year, the impact of centralizing surgical care may be more significant. In Sweden, before centralization, none of the centers handled less than five patients annually, which enabled these active centers to sustain high-quality care and surgical competence. As a result, centralization may have held lower impact in short-term outcomes than expected.

It is easy to oversimplify the benefits of centralization by assuming that higher case volumes automatically lead to greater surgical skill and fewer complications. However, even with an optimal pull-through procedure, the risk of impaired bowel function and HAEC remains significant [21–23]. In addition, between 4 and 30% of HSCR patients have associated developmental disorders or syndromes [16]. Therefore, the ability to manage the entire disease process is just as crucial as the ability to perform complex surgeries. Given this, multidisciplinary teams are essential in providing individualized, holistic care, and the conditions for developing such teams are better in high-volume hospitals [7].

Our results could not show major improvement nor deterioration in the diagnostic course, surgical management, and early postoperative outcome following centralization of care. The reported rise in HAEC rates is a point of observation but has many plausible explanations that do not necessarily relate to worsened outcome.

Further research is needed to assess long-term impact of centralization of HSCR. Studies addressing bowel function, quality of life, and patient and parent satisfaction following centralization are required. There is also a need for international studies with larger cohorts to counteract statistical uncertainty as well as research into multiple aspects of centralized care. Furthermore health-economic analysis and studies looking at the combined total effect of centralization of multiple diagnoses are required. This to secure that centralized care results in better health outcomes, better use of resources, increased competence of surgeons and better conditions for research, education and quality development with regards to these diagnoses.

### Conclusion

Centralization of care for HSCR does not seem to delay time to pull-through nor reduce severe complications, unplanned procedures under general anesthesia or readmissions up to 90 days after pull-through. There was an increase in HAEC following centralization, possibly due to increased awareness of mild HAEC.

## Data Availability

Data is provided within the manuscript.

## Abbreviations

HSCR: Hirschsprung’s disease
HAEC: Hirschsprung-associated enterocolitis
